# Supplementary material for: Beta spectral power during sleep is associated with impaired recall of extinguished fear
Source: Sleep. 2023 Aug 5;46(10):zsad209. doi: 10.1093/sleep/zsad209 (PMC10566240; doi:10.1093/sleep/zsad209)
Supplement: zsad209_suppl_Supplementary_Material [file zsad209_suppl_supplementary_material.docx]

**Supplementary materials**

**Beta spectral power during sleep is associated with impaired recall of extinguished fear**

Dan Denis^1*^, Ryan Bottary^2^, Tony J. Cunningham^3,4^, Sean P.A. Drummond^5^, Laura D. Straus^6,7^

^1^ Department of Psychology, University of York, York, United Kingdom

^2^ Institute for Graduate Clinical Psychology, Widener University, Chester, PA, USA

^3^ Center for Sleep and Cognition, Psychiatry Department, Beth Israel Deaconess Medical

Center, Boston, MA, USA

^4^ Division of Sleep Medicine, Harvard Medical School, Boston, MA, USA

^5^ School of Psychological Sciences, Turner Institute for Brain and Mental Health, Monash University, Clayton, Victoria, Australia

^6^ Mental Health Service, San Francisco Veterans Affairs Medical Center, San Francisco, CA, USA

^7^ Department of Psychiatry, University of California, San Francisco, CA, USA

* Corresponding author

Dan Denis

Room PS/B213,

Department of Psychology,

University of York,

York,

YO10 5DD

dan.denis@york.ac.uk

|  | Baseline | | | | Night 1 | | | | | Night 2 | | | | |
| --- | --- | --- | --- | --- | --- | --- | --- | --- | --- | --- | --- | --- | --- | --- |
|  | Normal sleep | Pre-ext dep | Post-ext dep | PTSD | | Normal sleep | Pre-ext dep | Post-ext dep | PTSD | | Normal sleep | Pre-ext dep | Post-ext dep | PTSD |
| Total sleep time (min) | 460 (23) | 463 (42) | 464 (40) | 365 (69) | | 473 (28) | - | 461 (40) | 399 (61) | | 454 (37) | 496 (30) | - | 395 (59) |
| Sleep onset latency (min) | 13.5 (17) | 12.3 (10.7) | 13.9 (12.6) | 23.1 (31.2) | | 10.4 (4.9) | - | 12.6 (11.3) | 14.4 (23.1) | | 14.7 (7.9) | 4.1 (3.6) | - | 14.2 (10.9) |
| Sleep efficiency (%) | 91 (7.6) | 92 (6.5) | 92 (3.7) | 85 (12.7) | | 93 (3.9) | - | 92 (2.8) | 92.7 (4.8) | | 89 (6.3) | 97 (1.5) | - | 91.3 (4.1) |
| Wake after sleep onset (min) | 34.5 (22.8) | 28.3 (32.7) | 27.2 (19.7) | 43.7 (51.2) | | 26.7 (19.2) | - | 28.8 (15.4) | 18.3 (12.1) | | 42.4 (33.4) | 10.1 (6.2) | - | 24.7 (19.9) |
| N1 (min) | 34.6 (13.1) | 33 (16.7) | 35.4 (16.3) | 33.3 (22.6) | | 36.8 (9.9) | - | 37 (15.1) | 24.2 (10.9) | | 43 (13.5) | 18.8 (9.7) | - | 24.5 (10.8) |
| N2 (min) | 239 (40) | 229 (31) | 227 (38.5) | 196 (43) | | 233 (30.6) | - | 222 (44) | 211 (39.6) | | 220 (29) | 227 (34.1) | - | 206 (39.4) |
| N3 (min) | 77.6 (24.6) | 87.2 (34.9) | 90.9 (37.4) | 65.9 (32.7) | | 80.2 (17.1) | - | 92.6 (36.3) | 69.4 (42.2) | | 75 (19.8) | 113 (29.6) | - | 63.7 (34.3) |
| REM (min) | 109 (21) | 114 (30) | 111 (28) | 70 (34) | | 123 (20) | - | 110 (21) | 94 (23) | | 116 (26.5) | 117 (22) | - | 101 (24.7) |
| N1 (% of TST) | 7.6 (3) | 7.1 (3.5) | 7.7 (3.6) | 9 (5.4) | | 7.8 (1.9) | - | 8.1 (3.4) | 6.2 (3.1) | | 9.5 (3.1) | 3.7 (1.9) | - | 6.1 (2.2) |
| N2 (% of TST) | 51.6 (5.3) | 49.7 (6.7) | 49 (7.2) | 53.7 (8.4) | | 49.2 (4.6) | - | 47.9 (7.7) | 53.4 (8.8) | | 48.5 (5.3) | 45.8 (6.1) | - | 52.3 (8.8) |
| N3 (% of TST) | 17 (5.2) | 18.8 (7.1) | 19.7 (8.4) | 18.4 (9.9) | | 17 (3.8) | - | 20.2 (8.3) | 17 (9.7) | | 16.5 (4.2) | 26.9 (6.2) | - | 15.9 (7.7) |
| REM (% of TST) | 23.9 (4.5) | 24.5 (5.6) | 23.7 (4.9) | 18.9 (7) | | 26.1 (4.3) | - | 23.8 (4.3) | 23.4 (3.6) | | 25.5 (5.3) | 23.5 (4) | - | 25.7 (5.8) |

**Table S1**. Sleep architecture for each night

*Note.* Sleep efficiency was calculated as the percentage of the sleep period (time between sleep onset and final awakening) spent asleep. All values displayed are mean (standard deviation).
